# Supplementary material for: Magnesium hexacyanoferrate nanocatalysts attenuate chemodrug-induced cardiotoxicity through an anti-apoptosis mechanism driven by modulation of ferrous iron
Source: Nat Commun. 2022 Dec 15;13:7778. doi: 10.1038/s41467-022-35503-y (PMC9755285; doi:10.1038/s41467-022-35503-y)
Supplement: Supplementary file 1 — Supplementary Information [file 41467_2022_35503_MOESM1_ESM.docx]

**Supplementary Information**

**Magnesium Hexacyanoferrate Nanocatalysts Attenuate Chemodrug-Induced Cardiotoxicity through an Anti-Apoptosis Mechanism Driven by Modulation of Ferrous Iron**

Minfeng Huo^1,2,3^, Zhimin Tang^4,5^, Liying Wang^1^, Linlin Zhang^2^, Haiyan Guo^6^, Yu Chen^7^, Ping Gu^3,4^* and Jianlin Shi^1,2,3^*

**Supplementary Figures**


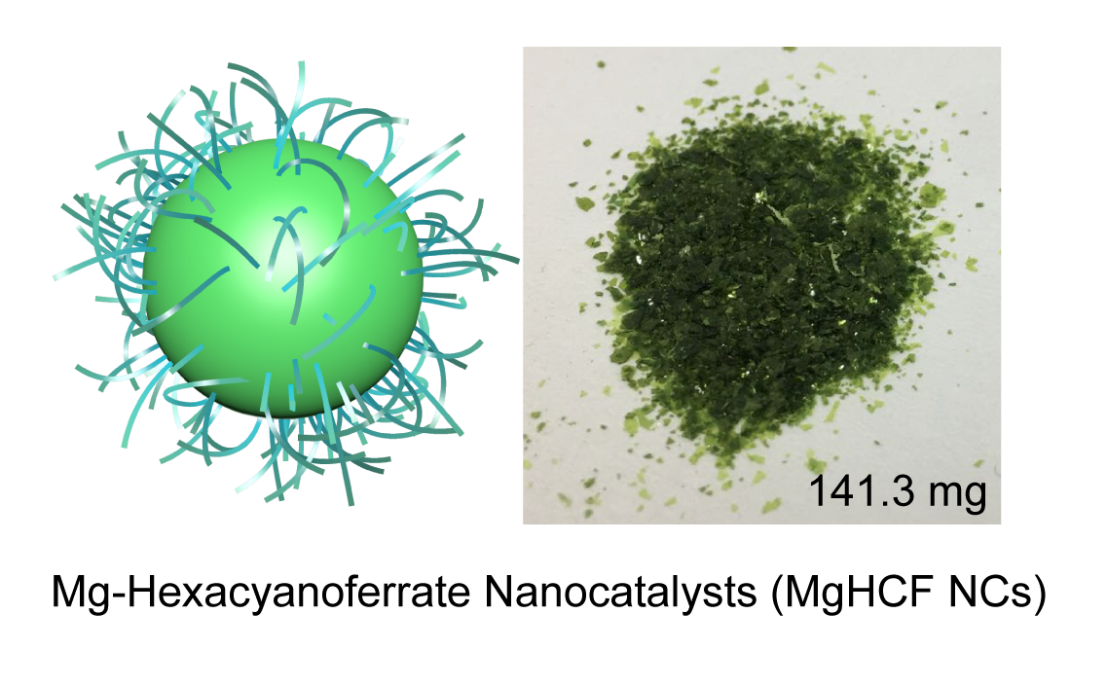


**Supplementary Figure 1.** MgHCF NCs powders in one synthesis.





**Supplementary Figure 2.** Nanoparticulate diameter distribution profile of MgHCF NCs calculated from 100 particles in the TEM image of **Figure 2b**.


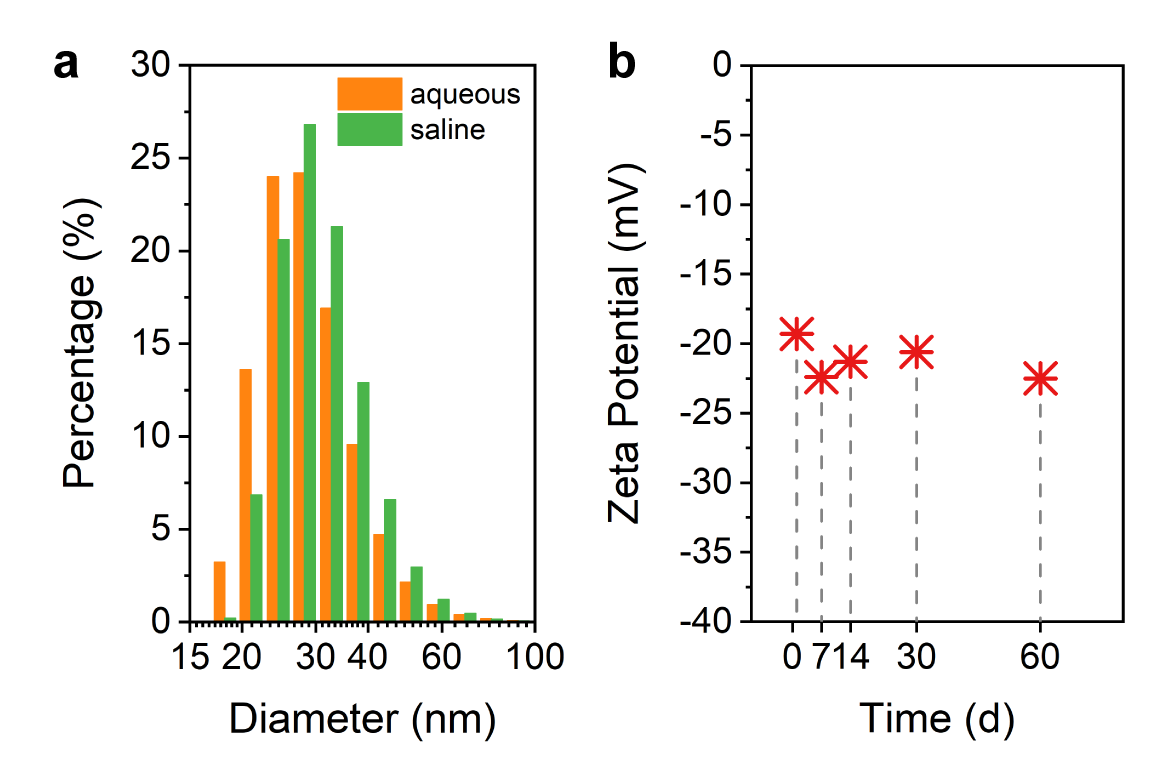


**Supplementary Figure 3. a**, Hydrodynamic diameter profile of MgHCF NCs suspended in water and saline. **b**, Zeta potential profile for MgHCF NCs suspended in water assayed at Day 0, 7, 14, 30 and 60.


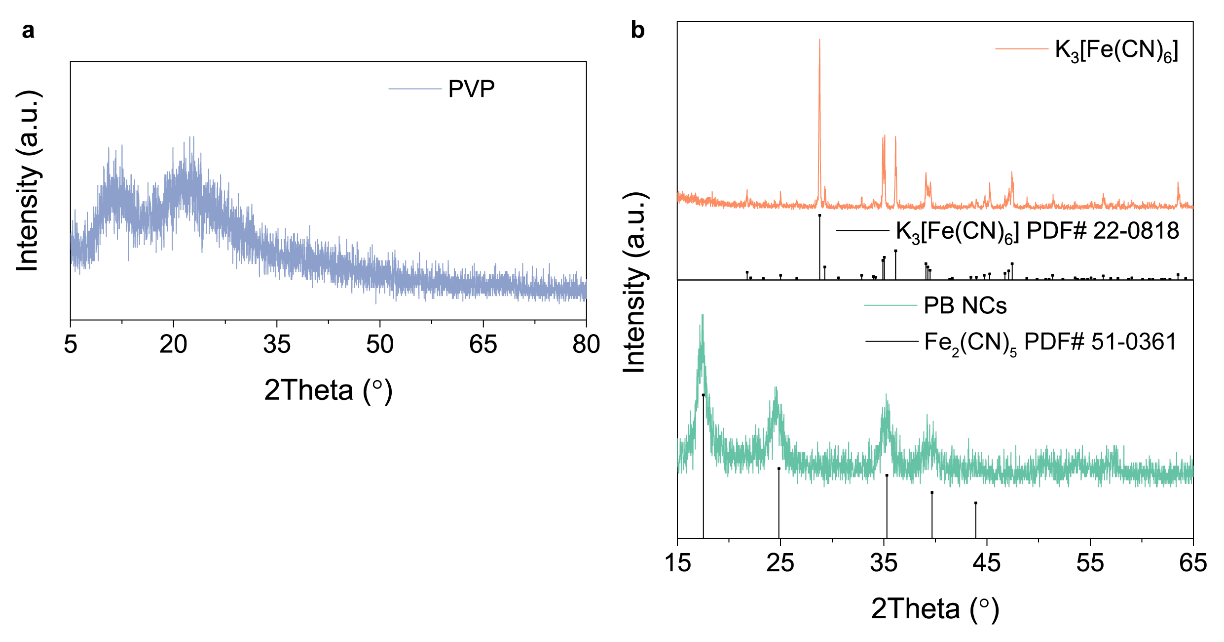


**Supplementary Figure 4. a**, XRD pattern of PVP. **b**, XRD patterns of K_3_[Fe(CN)_6_], reference PDF# 22-0818, synthetic PB NCs and reference PDF# 51-0361.





**Supplementary Figure 5.** Ion concentration profiles for Mg^2+^ and Fe^2+^ from MgHCF NCs incubated with RPMI complete media with fetal bovine.


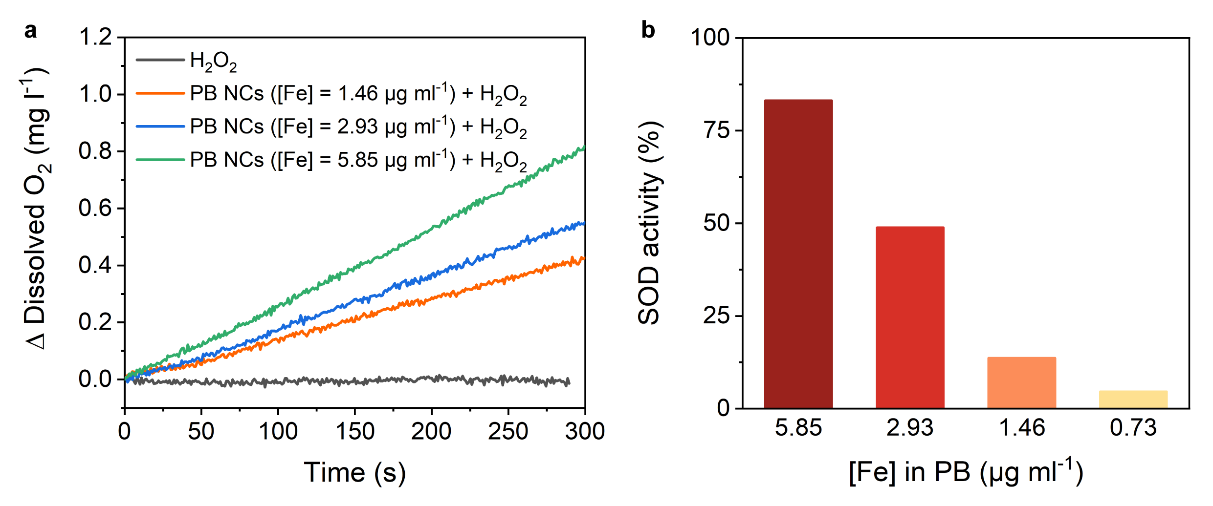


**Supplementary Figure 6. a**, Dissolved oxygen curves for PB NPs with different concentrations upon co-incubation with H_2_O_2_. **b**, SOD activity of PB NCs assayed with the SOD kit.


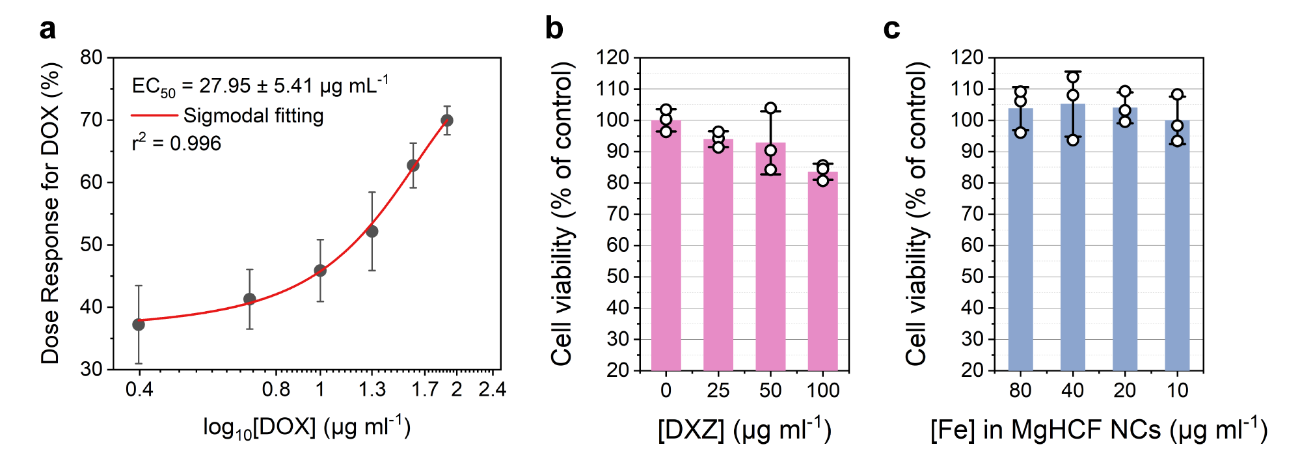


**Supplementary Figure 7. a**, Dose response cytotoxicity curves for DOX treated cardiomyocytes. **b-c**, Cytoviability profile for cardiomyocytes treated with varied concentrations of DXZ (**b**) and MgHCF NCs (**c**). n = 3. Data are presented as they are and mean ± s.d.


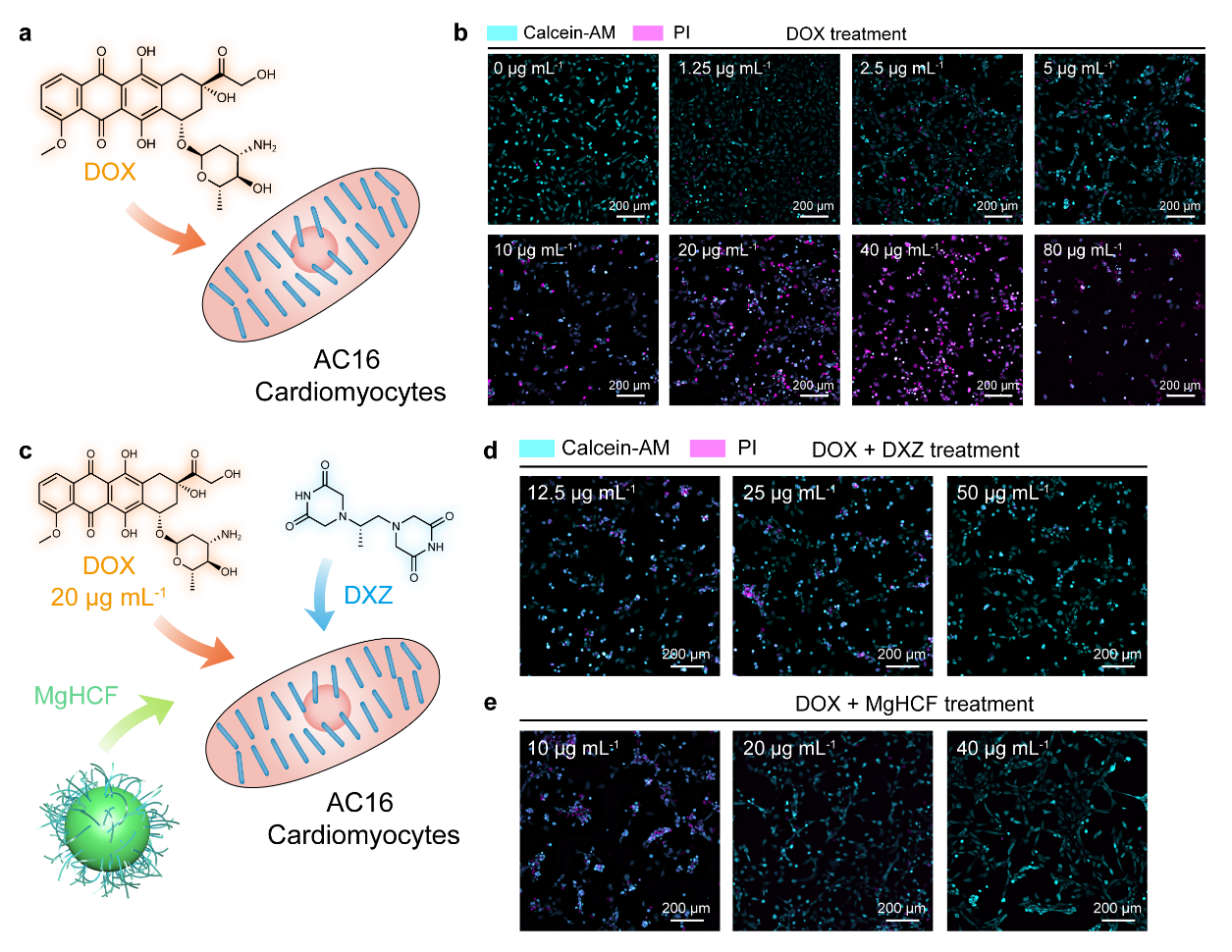


**Supplementary Figure 8. a**, Schematic illustration of the in vitro experiment of AC16 cell treatment with DOX. **b**, Confocal microscopic images of the Calcein-AM/PI dual stained AC16 cardiomyocytes treated with varied concentrations of DOX. **c**, Schematic illustration of the in vitro experiment of AC16 cell treatment with DOX + DXZ or DOX + MgHCF NCs. **d-e**, Confocal microscopic images of the Calcein-AM/PI dual stained DOX treated AC16 cardiomyocytes supplemented with varied concentrations of (**d**) DXZ and (**e**) MgHCF NCs.


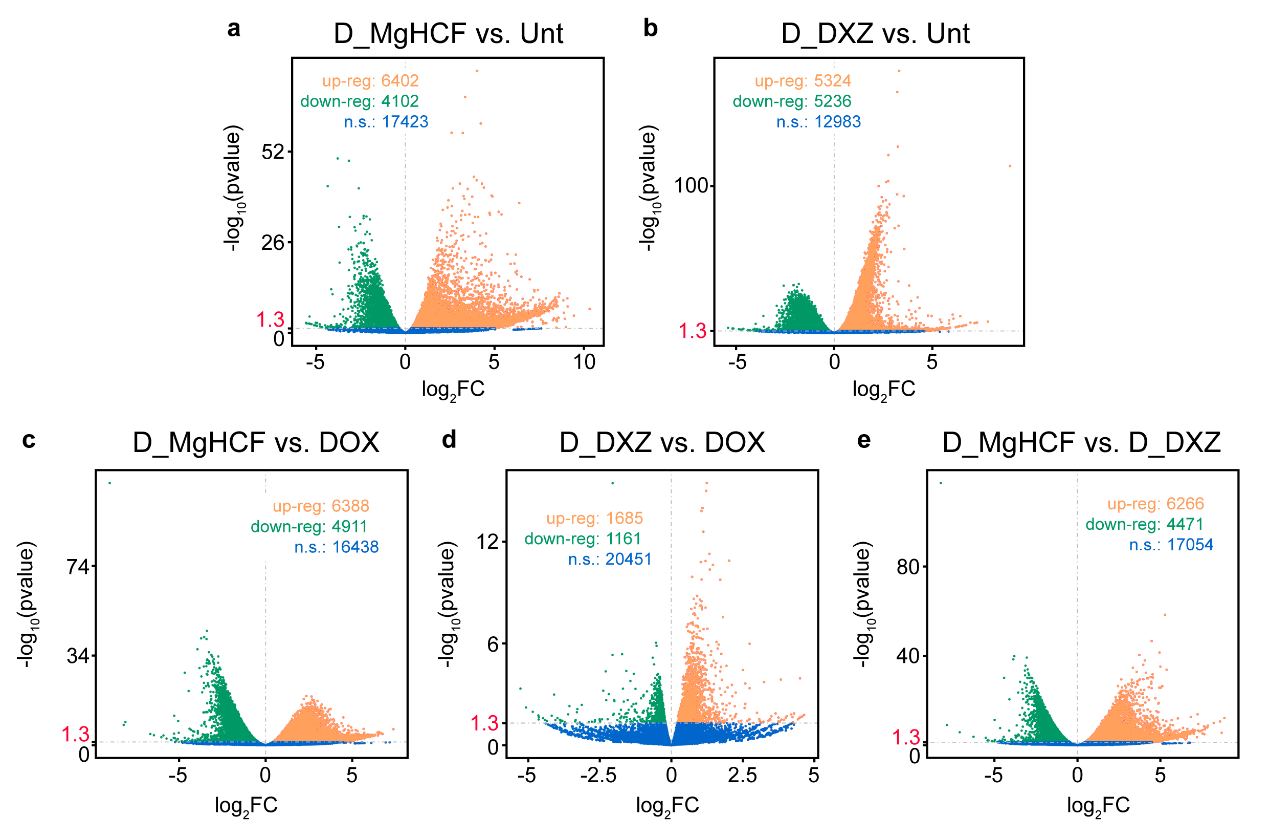


**Supplementary Figure 9.** Volcano plots for the DEG distribution between groups in a pairwise comparison. **a**, D_MgHCF vs. Unt; **b**, D_DXZ vs. Unt; **c**, D_MgHCF vs. DOX; **d**, D_DXZ vs. DOX and **e**, D_MgHCF vs. D_DXZ. Gene distribution data are presented as dots (orange for up-regulated, green for down-regulation and blue for non-significant). Significant tests are based on the negative binomial distribution. *P < 0.05 is equivalent to the -log_10_(pvalue) > 1.3.


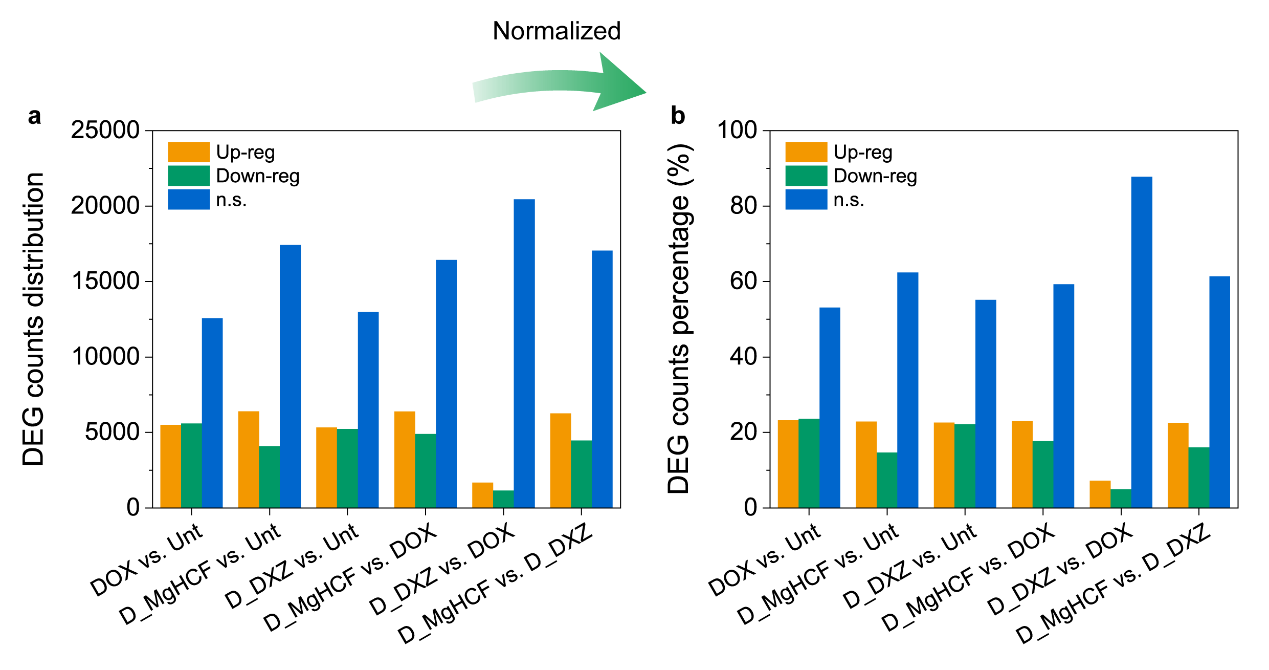


**Supplementary Figure 10.** Statistics for the DEG distribution between groups in a pairwise comparison. **a**, Primitive DEG counts distribution. **b**, Normalized DEG counts percentage distribution.


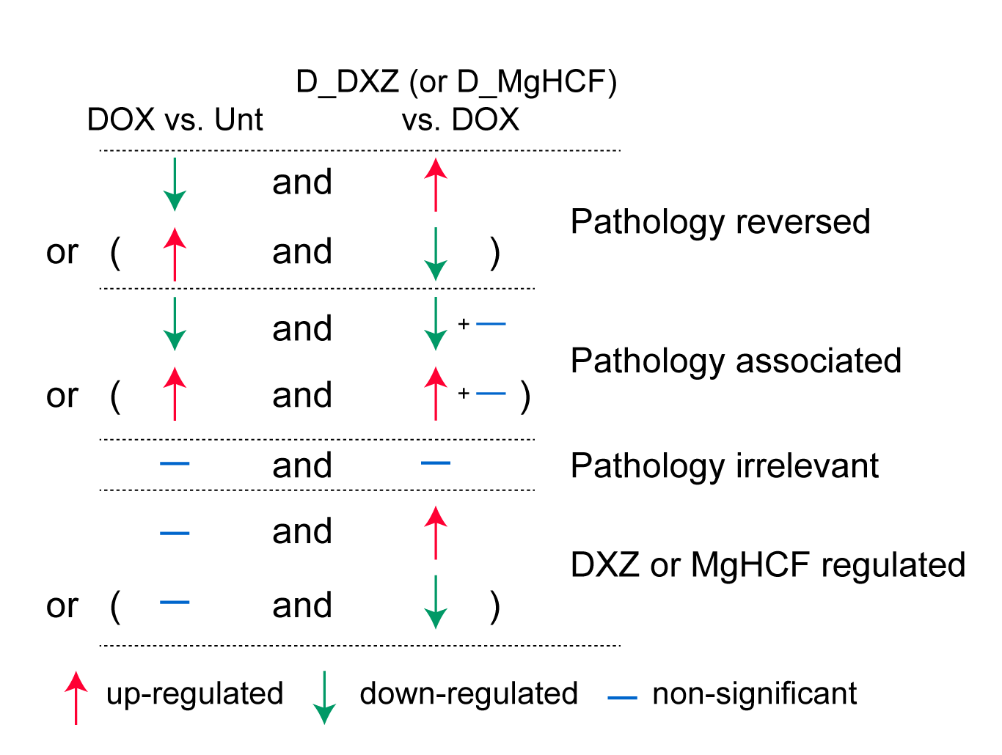


**Supplementary Figure 11.** Definition of the four gene groups of pathology-reversed, pathology-associated, pathology-irrelevant and DXZ- or MgHCF- regulated.


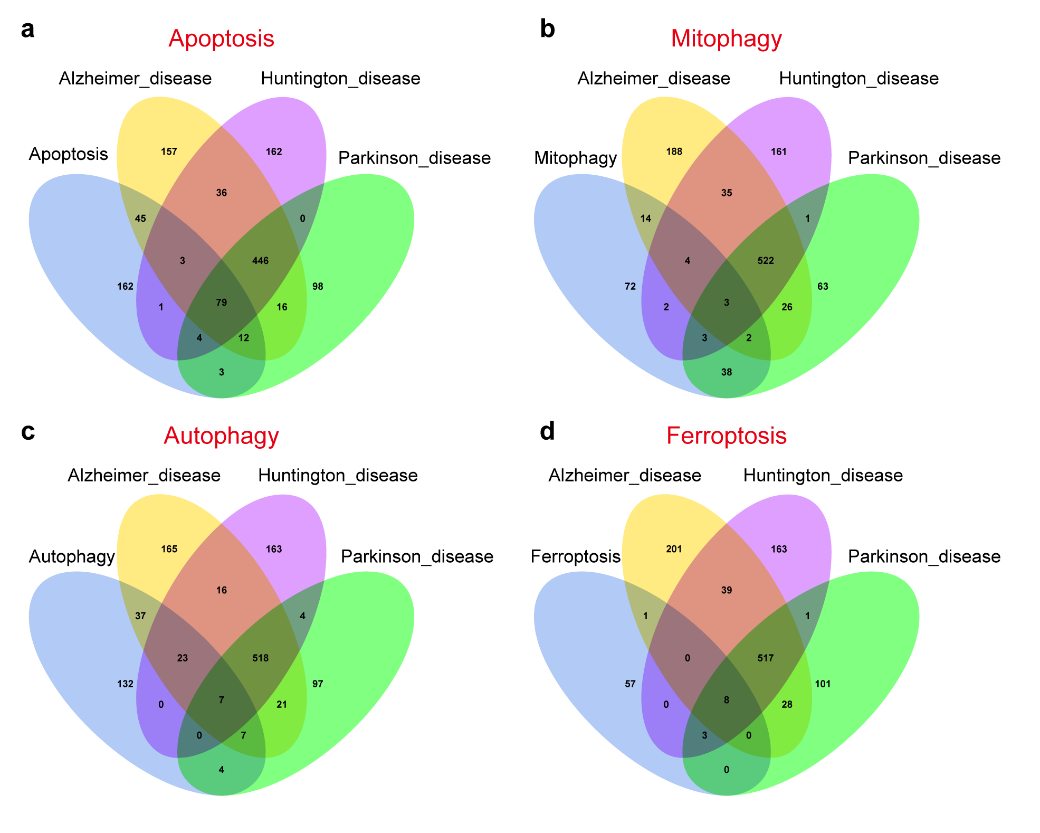


**Supplementary Figure 12.** Venn diagrams for the gene collections enriched in different KEGG pathways revealing the associations for Parkinson Disease, Huntington Disease and Alzheimer Disease to Apoptosis (a), Mitophagy (b), Autophagy (c) and Ferroptosis (d) respectively. Apoptosis (KEGG ID: mmu04210); Autophagy (KEGG ID: mmu04140); Mitophagy (KEGG ID: mmu04137); Ferroptosis (KEGG ID: mmu04216); Parkinson Disease (KEGG ID: mmu05012); Huntington Disease (KEGG ID: mmu05016) and Alzheimer Disease (KEGG ID: mmu05010).





**Supplementary Figure 13.** Gene count statistics for Parkinson disease, Alzheimer disease and Huntington disease intersected with apoptosis, autophagy, mitophagy and ferroptosis respectively.


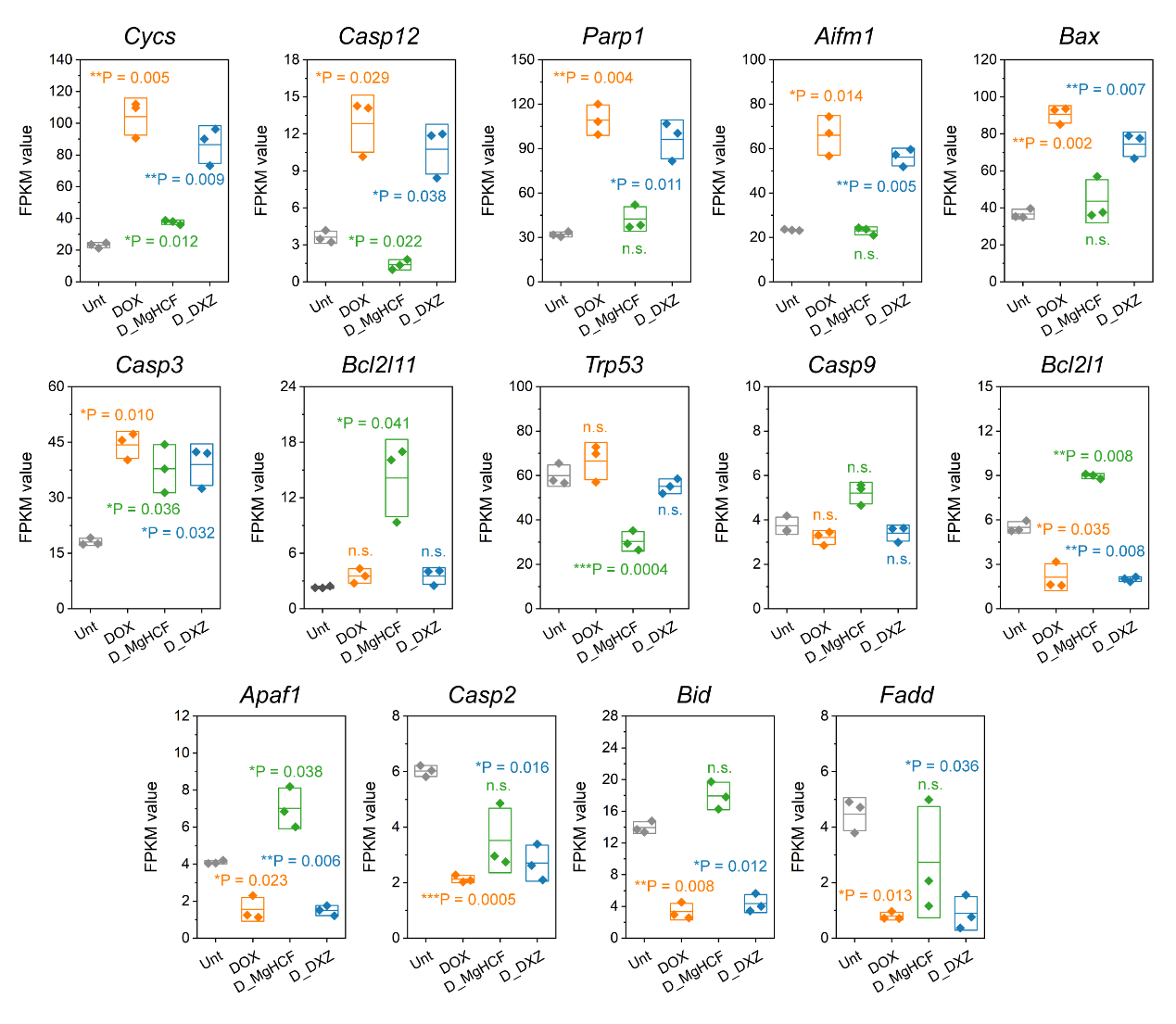


**Supplementary Figure 14.** FPKM values for selected mRNA expressions (*Cycs*, *Casp12*, *Parp1*, *Aifm1*, *Bax*, *Casp3*, *Bcl2l11*, *Trp53*, *Casp9*, *Bcl2l1*, *Apaf1*, *Casp2*, *Bid* and *Fadd*) for cells in Unt, DOX, D_MgHCF and D_DXZ groups. Significant analyses have been conducted for the Unt group by a student’s t test (paired two-tailed). P values are always indicated (*P < 0.05, **P < 0.01, ***P < 0.001, n.s. for non-significant). n = 3. Data are presented as they are and mean (line) ± s.d. (bounds of box).


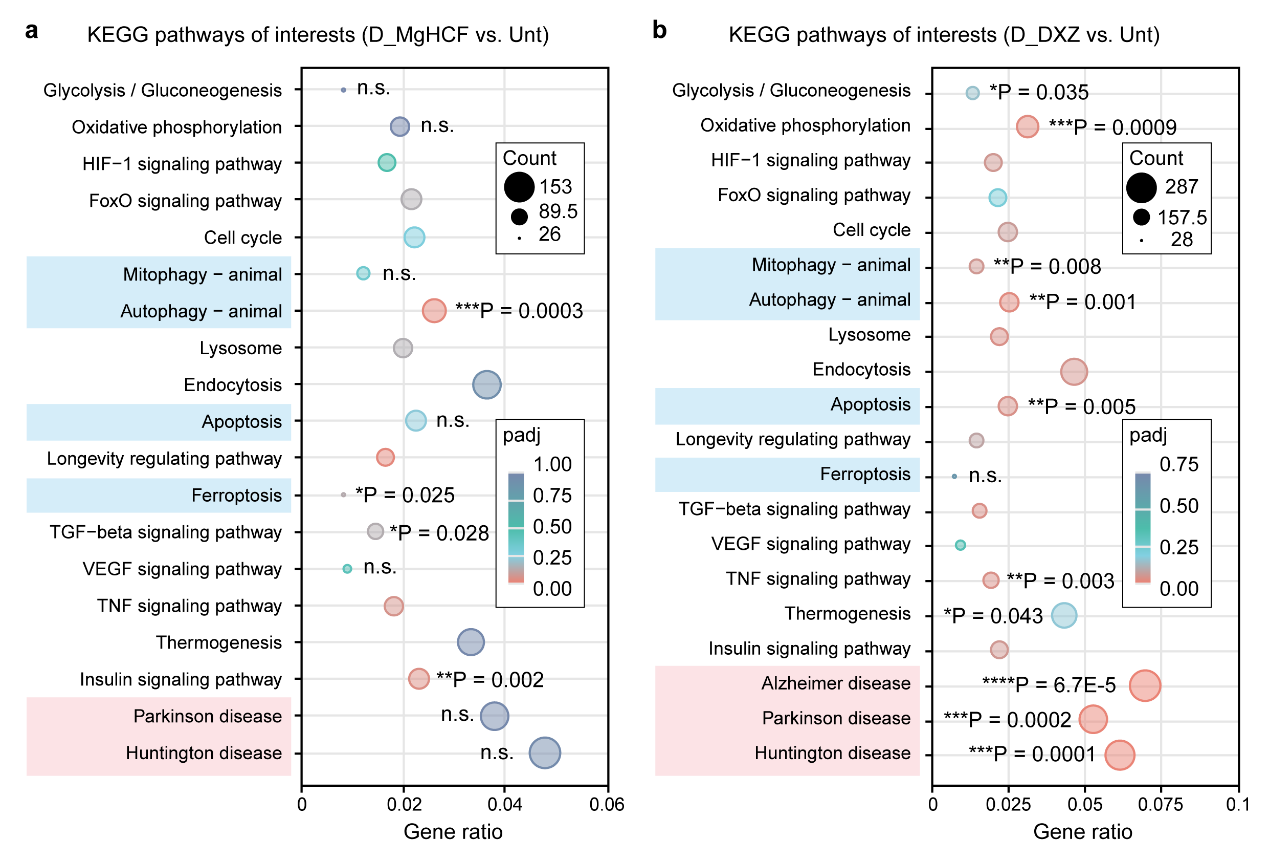


**Supplementary Figure 15.** KEGG enrichment pathways for cells in D_MgHCF (a) or D_DXZ group (b) as compared to the untreated group respectively. Significant tests (padj) are based on the negative binomial distribution with further corrections (Benjamini–Hochberg procedure). *P < 0.05, **P < 0.01, ***P < 0.001 and ****P < 0.0001.





**Supplementary Figure 16.** Quantification of the bands as relative expression to the β-actin for proteins of Cycs, Bax, Bid, Bcl-xl, c-Parp1, Aif and c-Casp3 (for cleaved casp3). n = 3. Data are presented as they are and mean ± s.d.


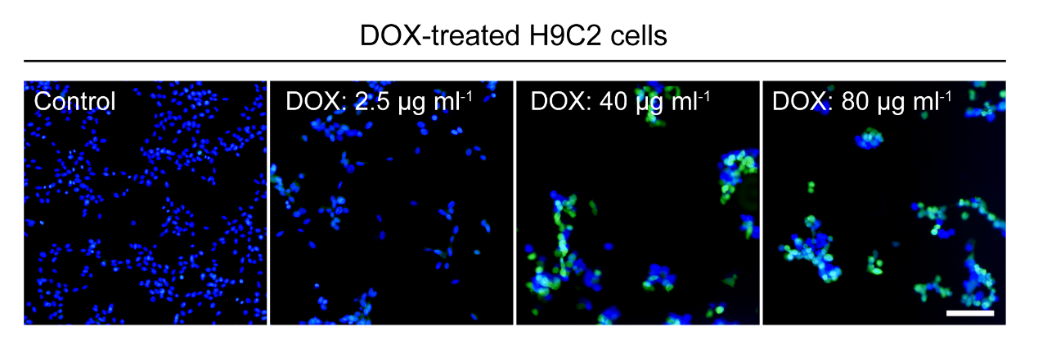


**Supplementary Figure 17.** Confocal microscopic images of DCFH-DA-stained cardiomyocytes treated with DOX at varied concentrations. Scale bar: 100 μm.


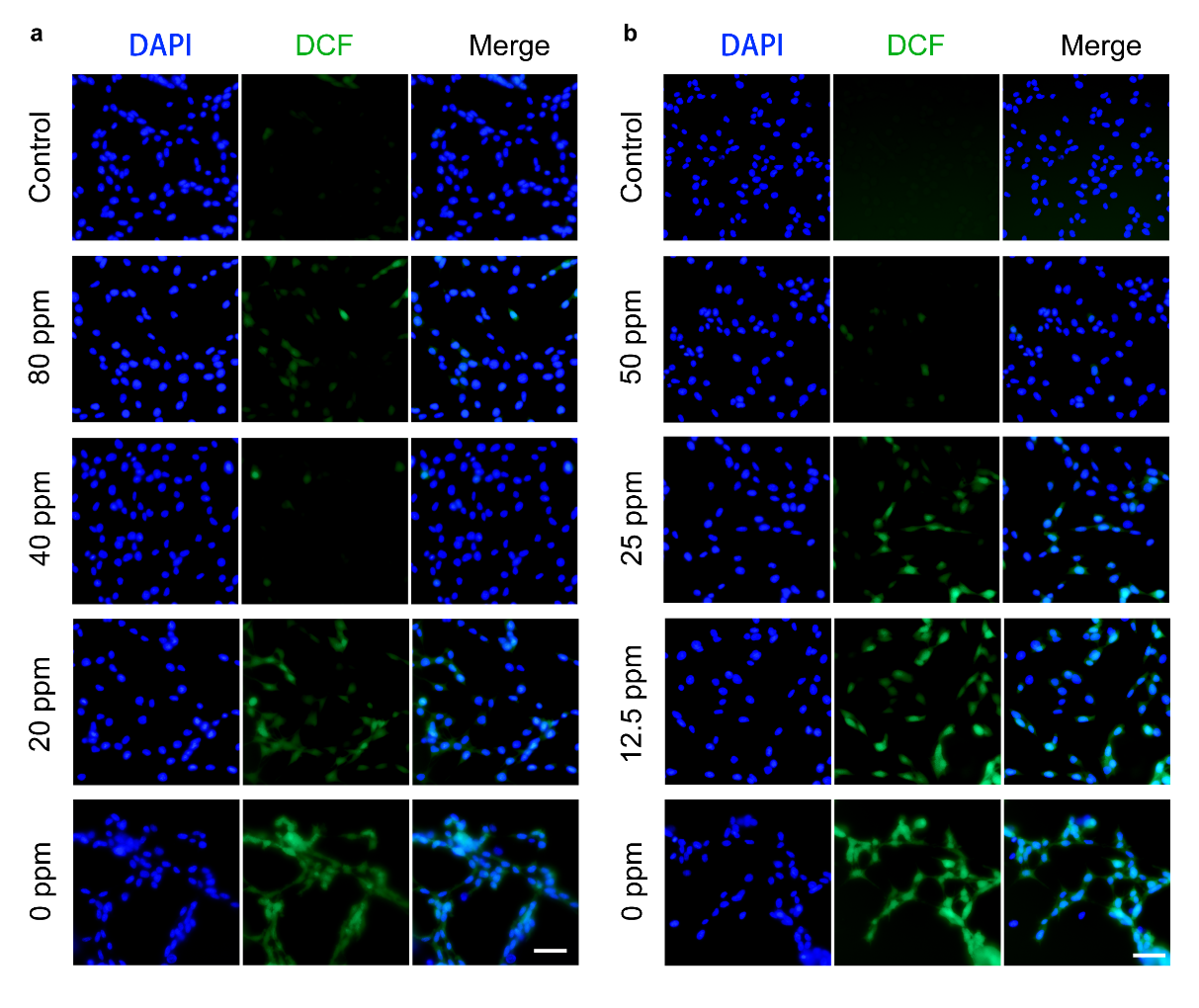


**Supplementary Figure 18. a-b,** Confocal microscopic images of DCFH-DA-stained cardiomyocytes treated with DOX supplemented with MgHCF (**a**) or DXZ (**b**) at varied concentrations. Scale bar: 50 μm.


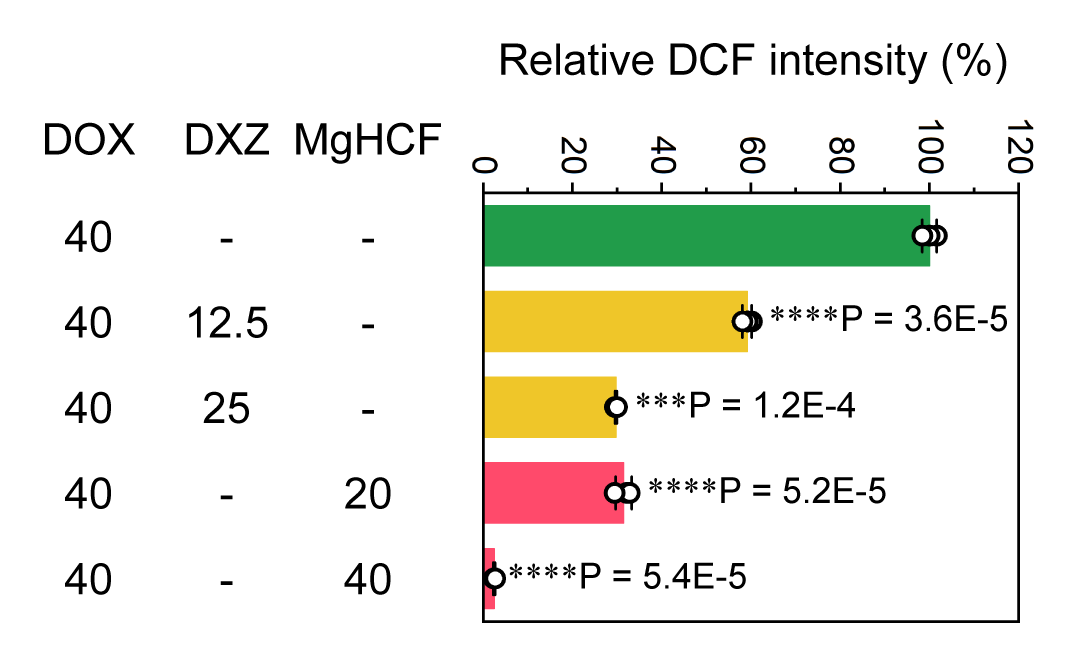


**Supplementary Figure 19.** Relative DCF fluorescence intensity of the Confocal microscopic images in Figure 5e. Data are presented as mean ± s.d. n = 3. Statistical significance is assessed by Student t’s one-tailed test. **P < 0.01, ***P < 0.001, ****P < 0.0001 and n.s. for non-significant.


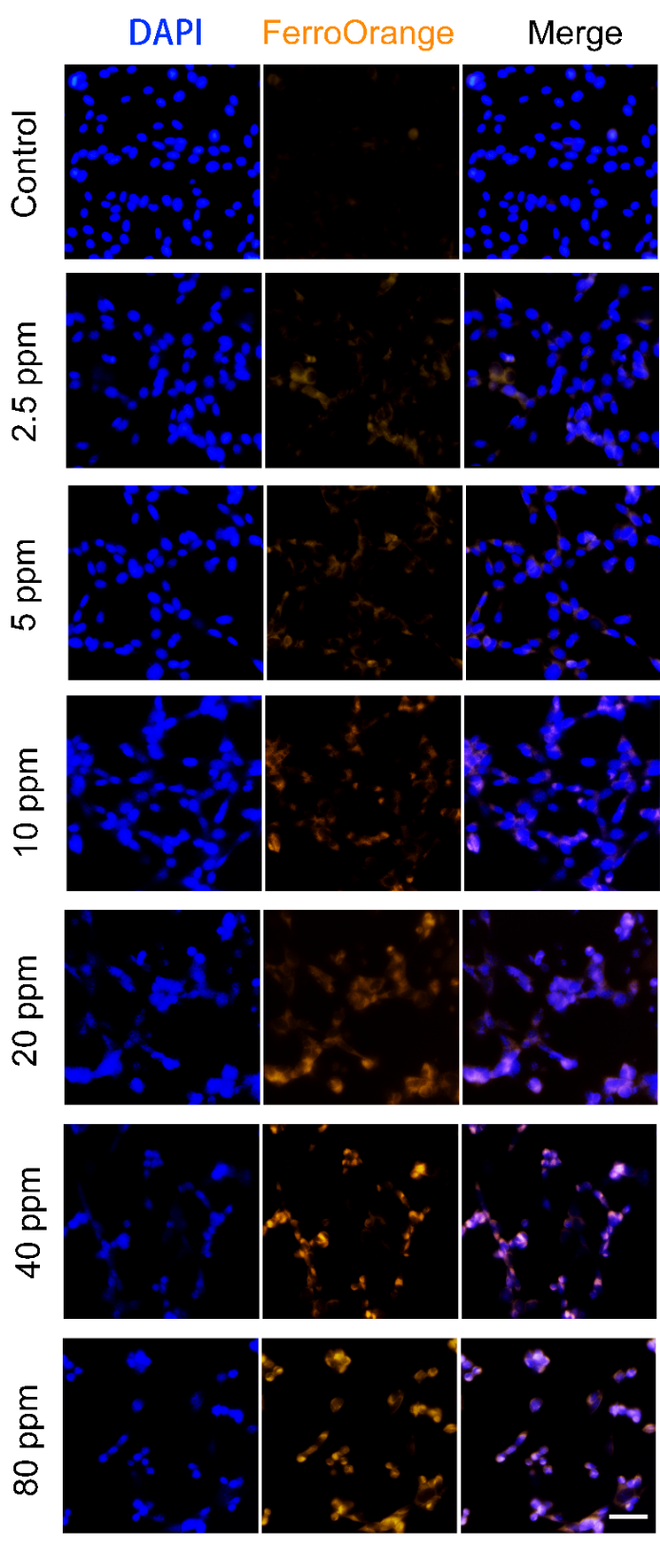


**Supplementary Figure 20.** Confocal microscopic images of FerroOrange-stained cardiomyocytes treated with DOX at varied concentrations. Scale bar: 50 μm.


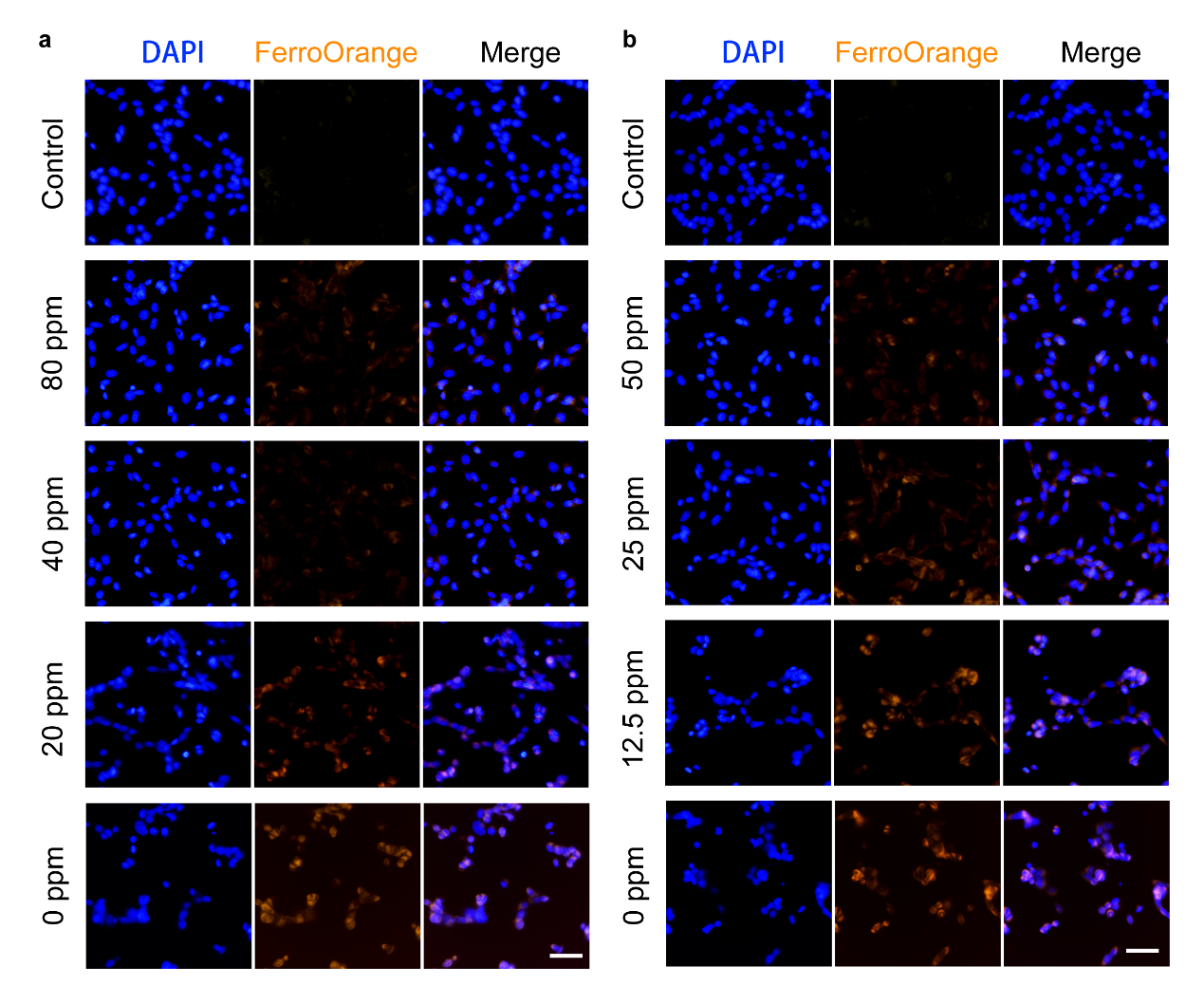


**Supplementary Figure 21. a-b,** Confocal microscopic images of FerroOrange-stained cardiomyocytes treated with DOX supplemented with MgHCF (**a**) and DXZ (**b**) at varied concentrations. Scale bar: 50 μm.


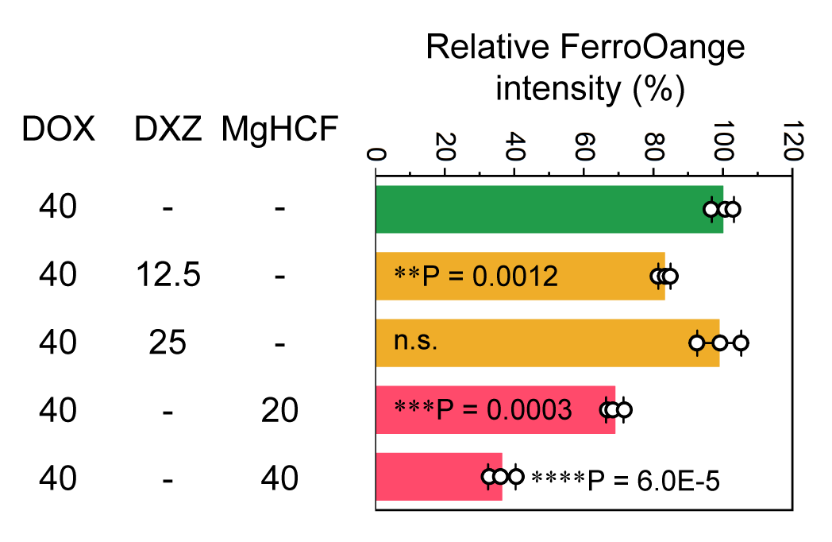


**Supplementary Figure 22.** Relative FerroOrange fluorescence intensity of the Confocal microscopic images in Figure 5f. Data are presented as mean ± s.d. n = 3. Statistical significance is assessed by Student t’s one-tailed test. **P < 0.01, ***P < 0.001, ****P < 0.0001 and n.s. for non-significant.


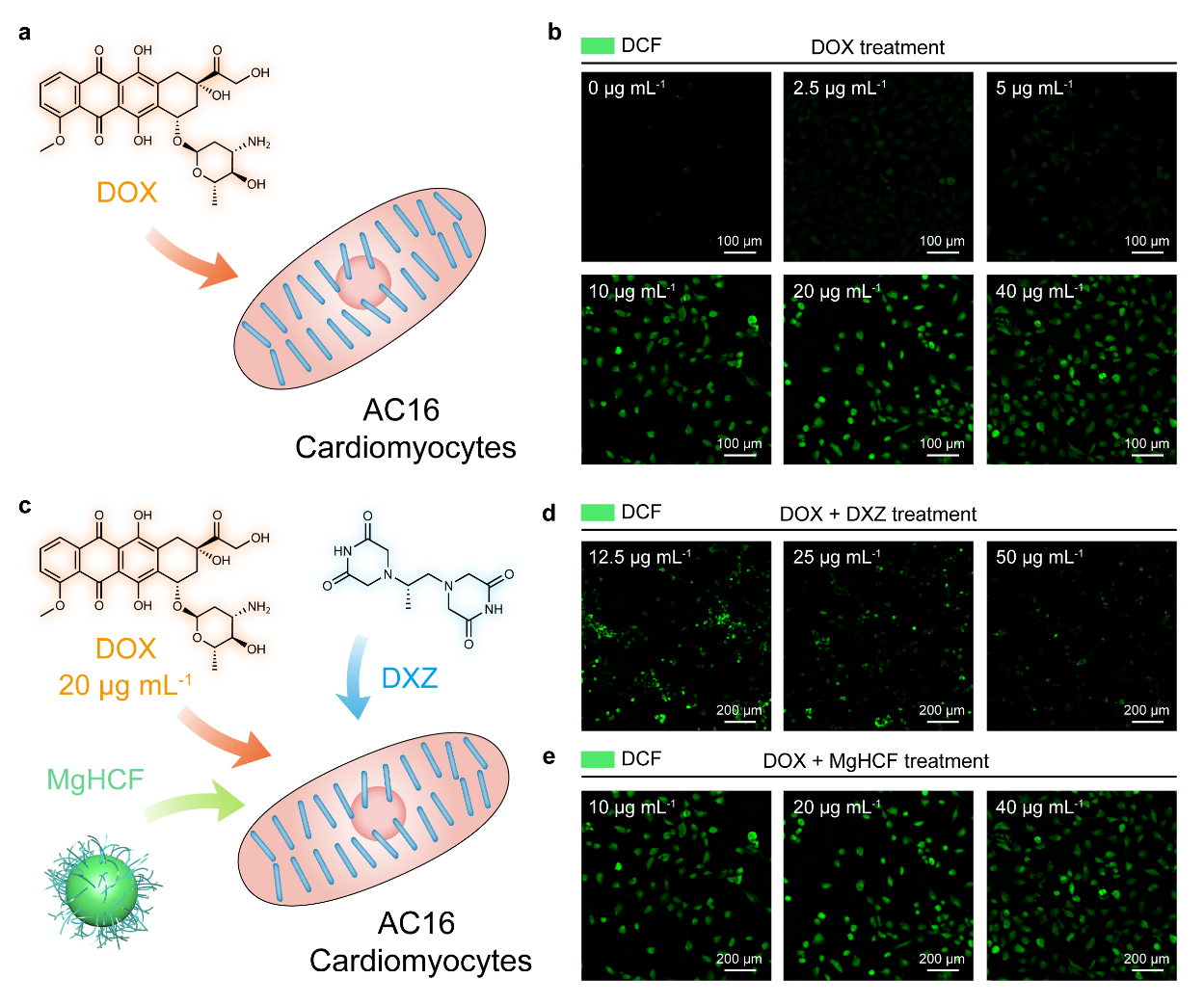


**Supplementary Figure 23. a**, Schematic illustration of the in vitro experiment of AC16 cell treatment with DOX. **b**, Confocal microscopic images of the DCFH-DA stained AC16 cardiomyocytes treated with varied concentrations of DOX. **c**, Schematic illustration of the in vitro experiment of AC16 cell treatment with DOX + DXZ or DOX + MgHCF NCs. **d-e**, Confocal microscopic images of DCFH-DA stained DOX treated AC16 cardiomyocytes supplemented with varied concentrations of (**d**) DXZ and (**e**) MgHCF NCs.


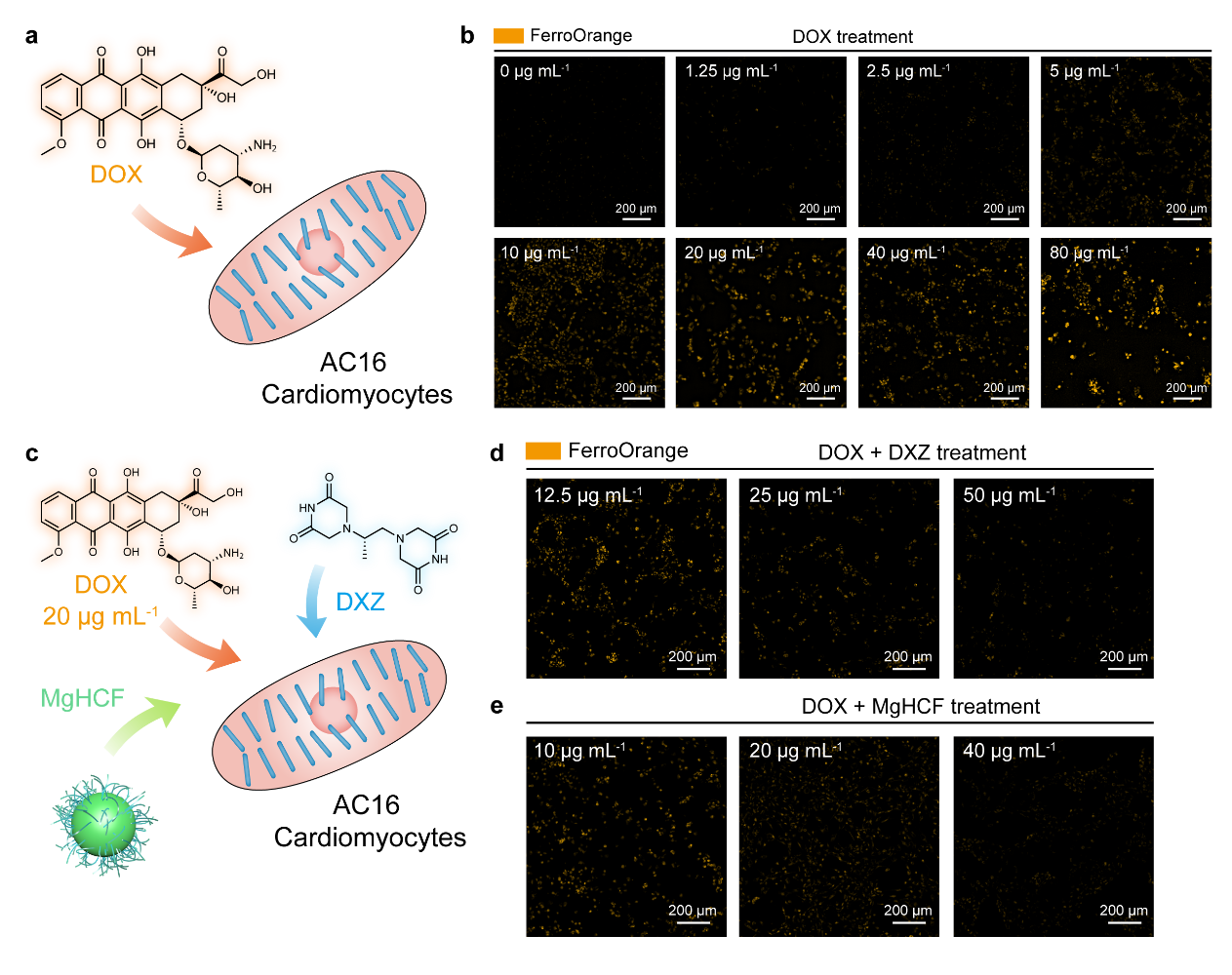


**Supplementary Figure 24. a**, Schematic illustration of the in vitro experiment of AC16 cell treatment with DOX. **b**, Confocal microscopic images of the FerroOrange stained AC16 cardiomyocytes treated with varied concentrations of DOX. **c**, Schematic illustration of the in vitro experiment of AC16 cell treatment with DOX + DXZ or DOX + MgHCF NCs. **d-e**, Confocal microscopic images of FerroOrange stained DOX treated AC16 cardiomyocytes supplemented with varied concentrations of (**d**) DXZ and (**e**) MgHCF NCs.


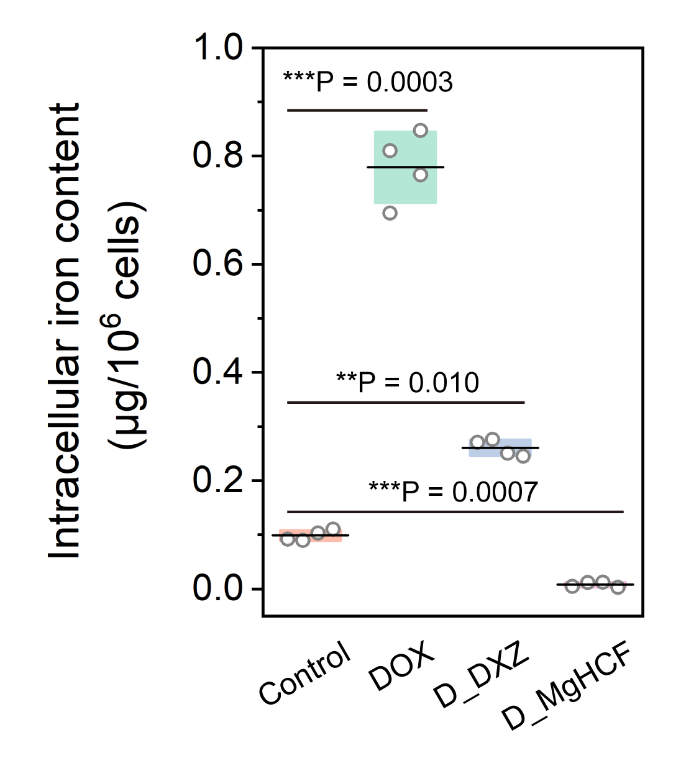


**Supplementary Figure 25.** Intracellular iron content of AC16 cells from different treatment groups. Data are presented as they are and mean ± s.d. P values are always indicated by a student’s t test (paired two-tailed) (**P < 0.01, ***P < 0.001, n.s. for non-significant). n = 4.


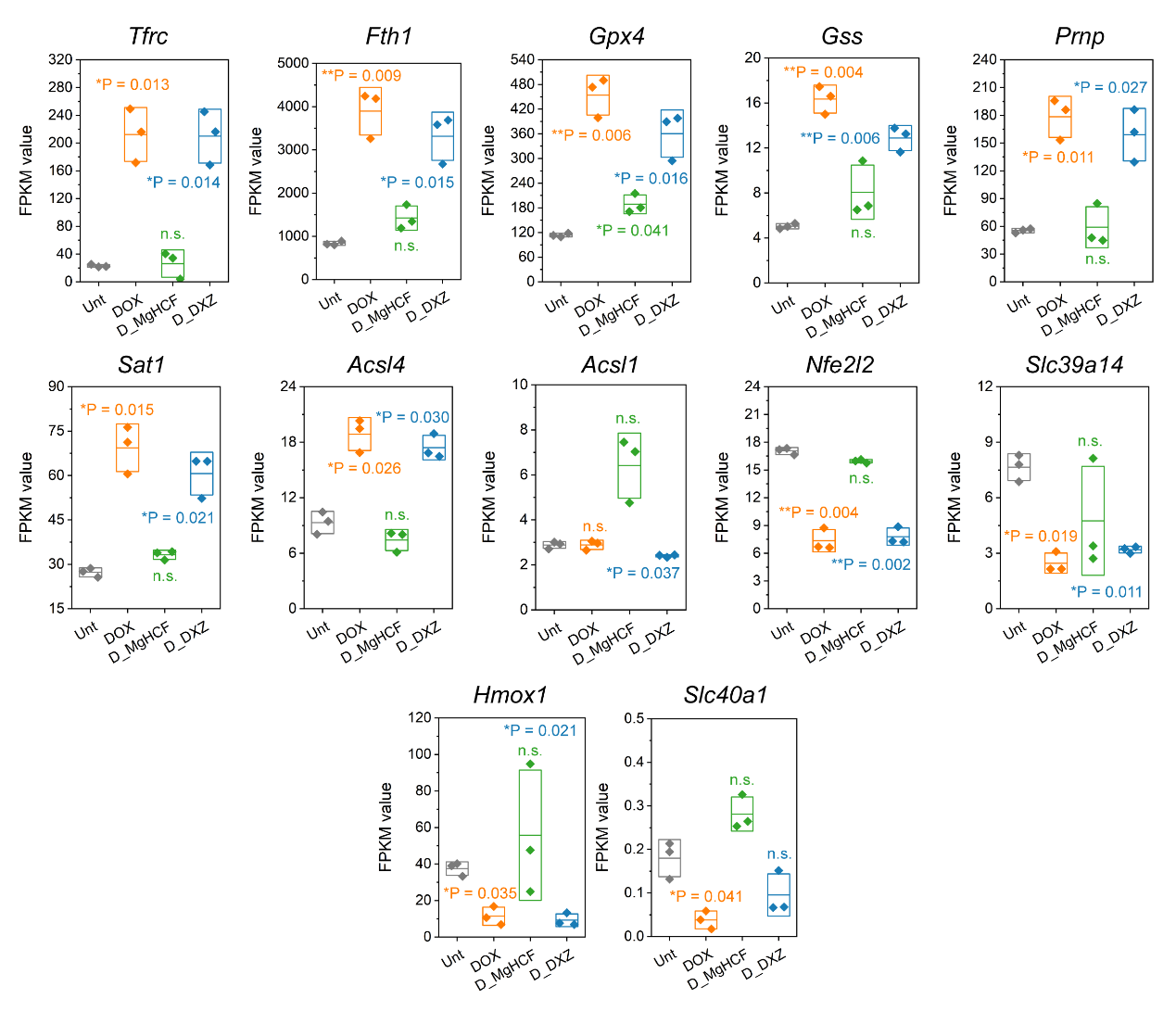


**Supplementary Figure 26.** FPKM values for selected mRNA expressions (*Tfrc*, *Fth1*, *Gpx4*, *Gss*, *Prnp*, *Sat1*, *Acsl4*, *Acsl1*, *Nfe2l2*, *Slc39a14*, *Hmox1* and *Slc40a1*) for cells in Unt, DOX, D_MgHCF and D_DXZ groups. Significant analyses have been conducted for the Unt group by a student’s t test (paired two-tailed). n = 3. P values are always indicated (*P < 0.05, **P < 0.01, ***P < 0.001, n.s. for non-significant). Data are presented as they are and mean (line) ± s.d. (bounds of box).





**Supplementary Figure 27.** Quantification of the bands as relative expression to the β-actin for proteins of Ho-1, Slc40a1, Tfrc, Nrf2 and Fth1. n = 3. Data are presented as they are and mean ± s.d.


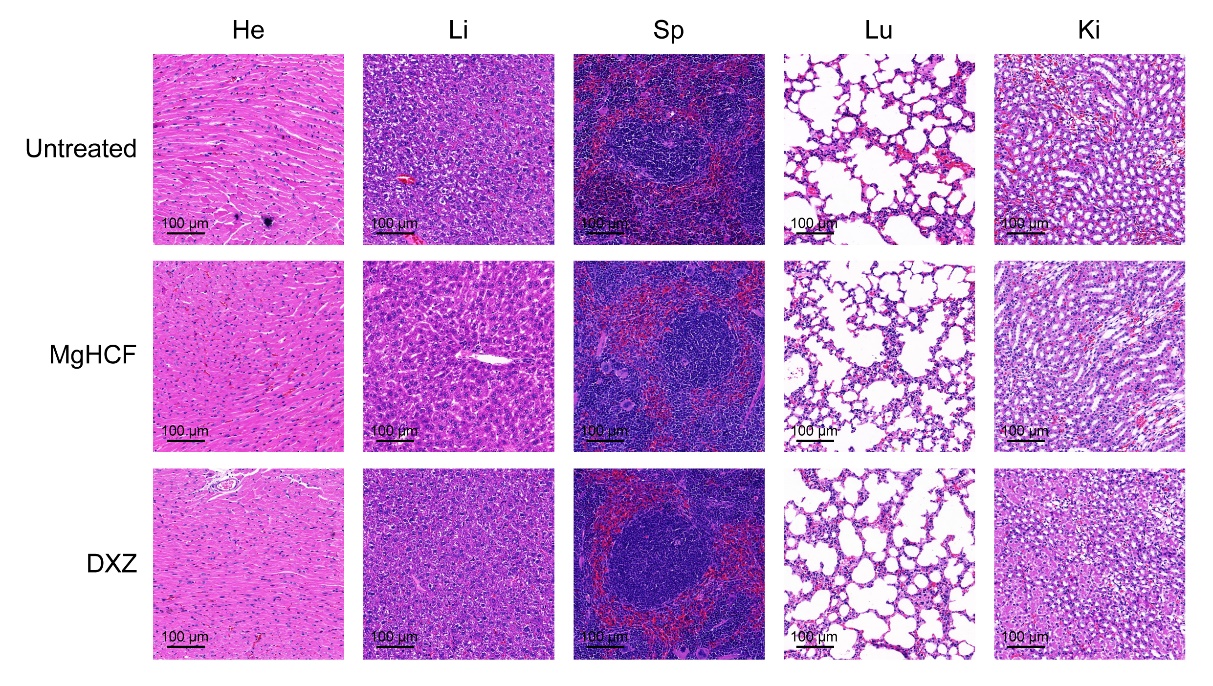


**Supplementary Figure 28.** H&E staining tissue sections of mice from different groups after the in vivo biocompatibility evaluation period.





**Supplementary Figure 29.** Main haematological routine indexes (WBC: white blood cells (10^9^ L^-1^); LYMPH: lymphocytes (10^9^ L^-1^); RBC: red blood cells (10^12^ L^-1^); HGB: hemoglobin (g L^-1^); MCV: mean corpuscular volume (fL); RDW: red cell distribution width (%)) of mice from different groups (Control, MgHCF and DXZ groups) after the in vivo biocompatibility evaluation period. n = 4. Data are presented as they are and mean ± s.d.


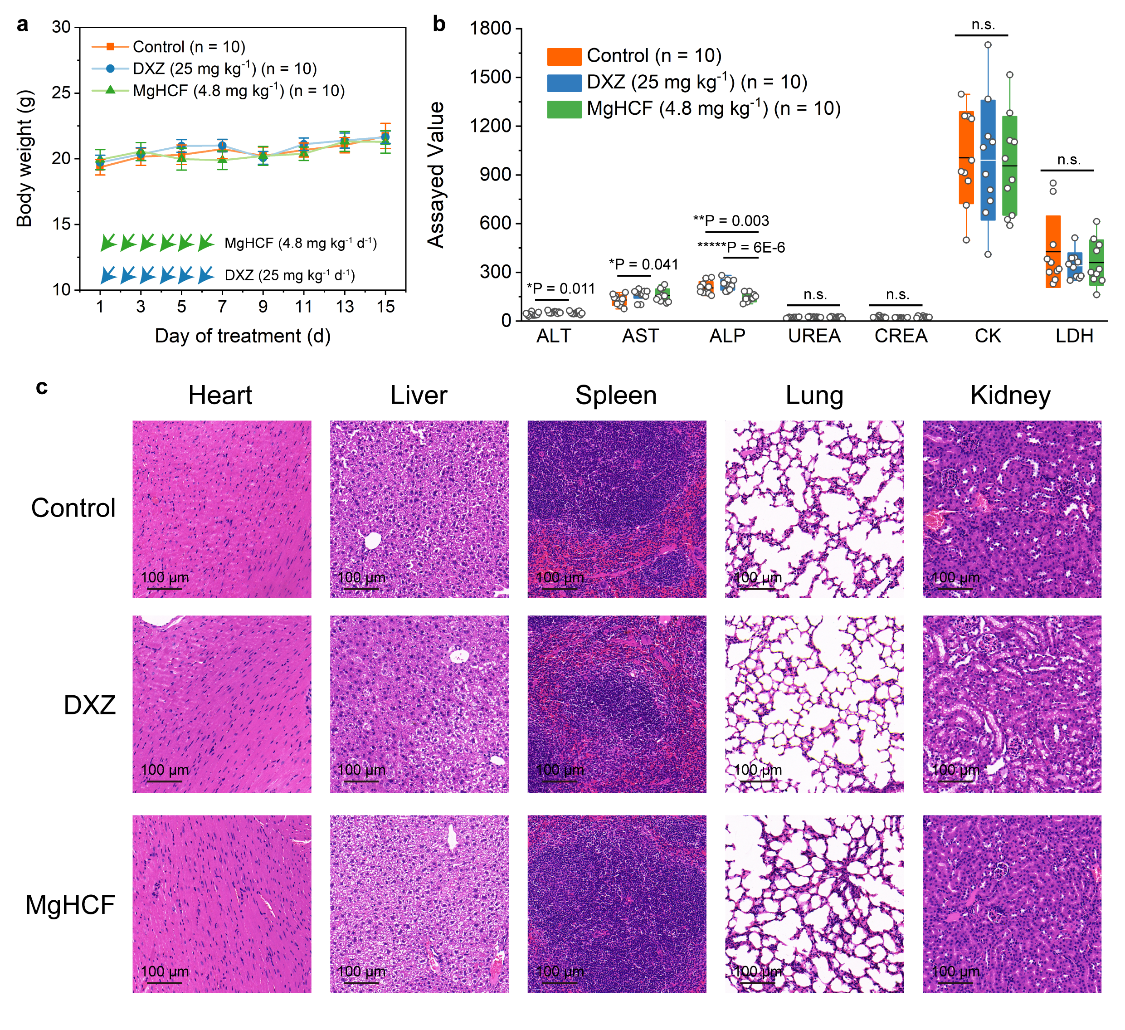


**Supplementary Figure 30. a,** Body weights profile of mice from different groups during the multiple dose biocompatibility evaluation. **b-c**, Major blood biochemical indexes (**b**) and H&E staining tissue sections (**c**) of mice from different groups at the end of the multiple dose biocompatibility evaluation. **a, b**, Data are presented as they are and mean ± s.d.. Statistical significance (to the control group) is assessed by Student t’s two-tailed test. *P < 0.05, **P < 0.01, *****P < 0.00001, n.s. for non-significant.

**
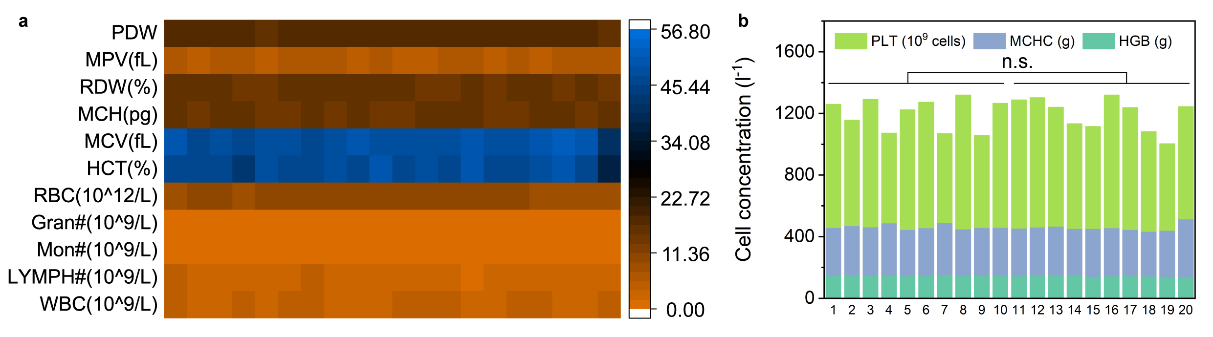
**

**Supplementary Figure 31.** Haematological routine indexes of mice from control and MgHCF group (n = 10) recorded at the end of the evaluation period of one month. **a**, WBC: white blood cells; LYMPH: lymphocytes; MON: monocytes; Gran (granulocyte); RBC: red blood cells; HCT: Hematocrit; MCV: mean corpuscular volume; MCH: Mean RBC hemoglobin content; RDW: RBC distribution width; MPV: Mean platelet volume and PDW: platelet distribution width. **b**, PLT: platelet; MCHC: mean corpuscular-hemoglobin concentration and HGB: hemoglobin. Data are presented as they are. Statistical significance (to the control group) is assessed by Student t’s two-tailed test. n.s. for non-significant.


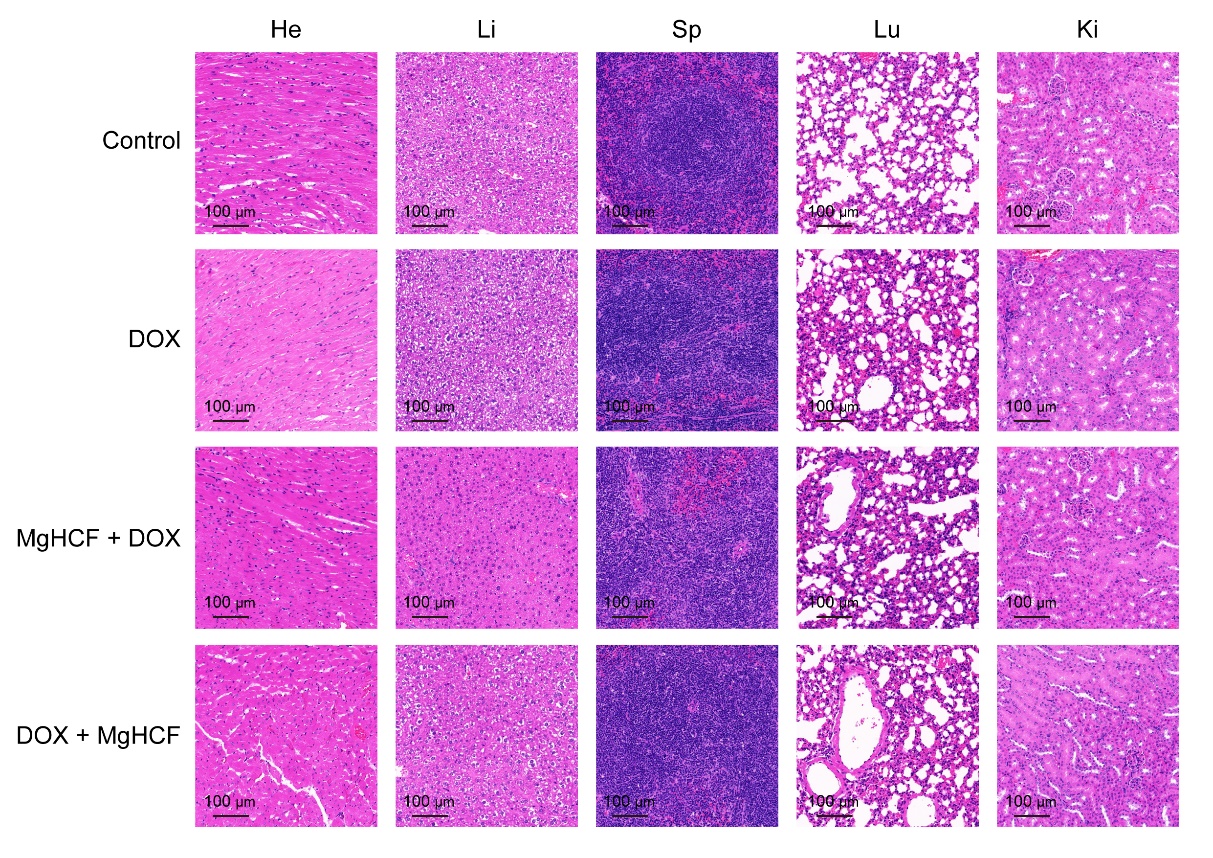


**Supplementary Figure 32.** H&E staining tissue sections of mice from different groups after the in vivo cardioprotective therapeutic evaluation period.





**Supplementary Figure 33.** Main haematological routine indexes (WBC, 10^9^ L^-1^; LYMPH, 10^9^ L^-1^; RBC, 10^12^ L^-1^; HGB, g L^-1^; MCV, fL; RDW, %) of mice from different groups (Control, DOX, MgHCF + DOX and DOX + MgHCF groups) after the in vivo cardioprotective evaluation period. n = 4. Data are presented as they are and mean ± s.d.


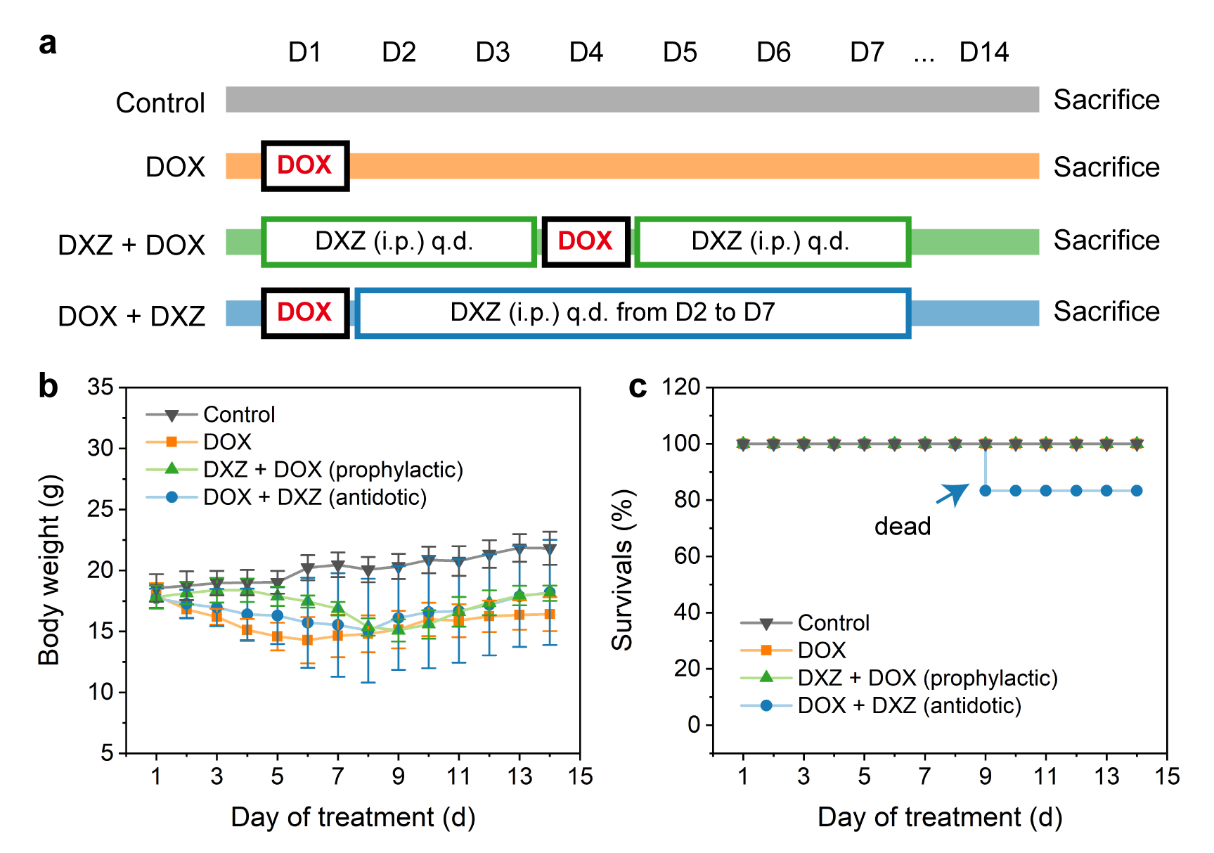


**Supplementary Figure 34. a**, Dosing schedules of the *in vivo* cardiac protection evaluation. **b, c**, Body weights (**b**) and survival rates (**c**) of mice from different groups during the evaluation period. n = 6. Data are presented as mean ± s.d..


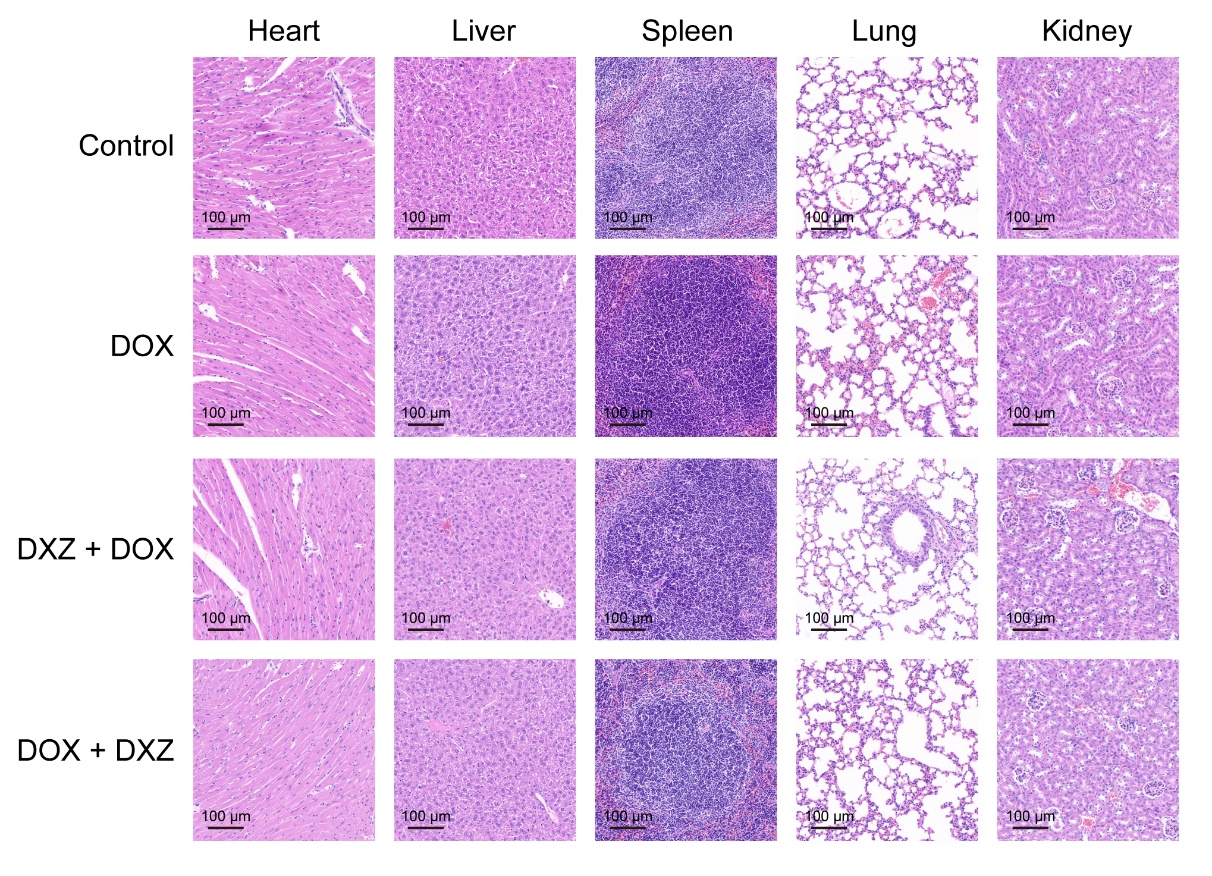


**Supplementary Figure 35.** H&E staining tissue sections of mice from different groups after the in vivo cardioprotective therapeutic evaluation period.


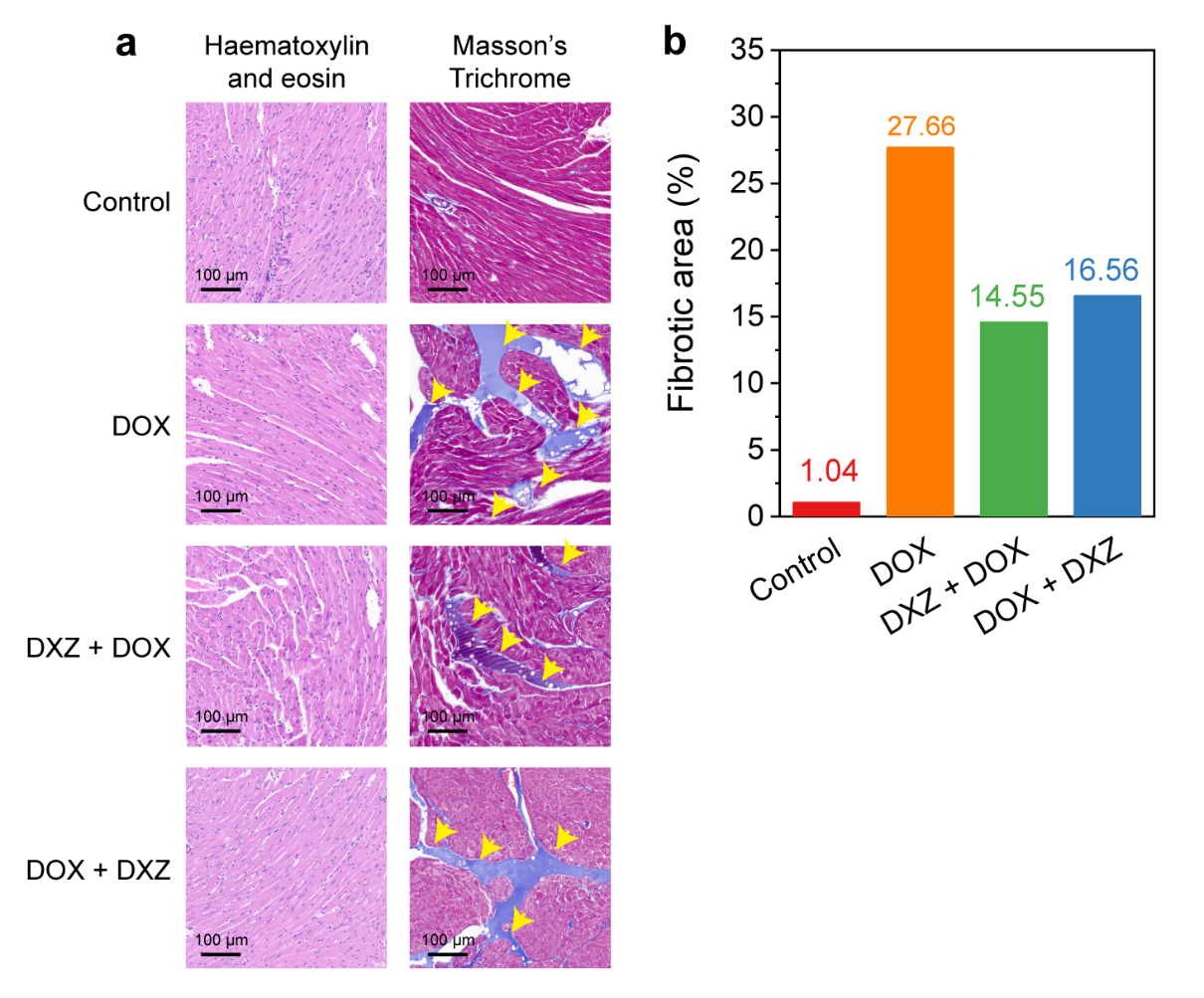


**Supplementary Figure 36. a**, H&E staining and Masson’s Trichrome staining images of the heart sections in different groups. **b**, Fibrotic zone percentages on the heart tissue of mice in different groups revealed by the Masson’s Trichrome staining imaging.


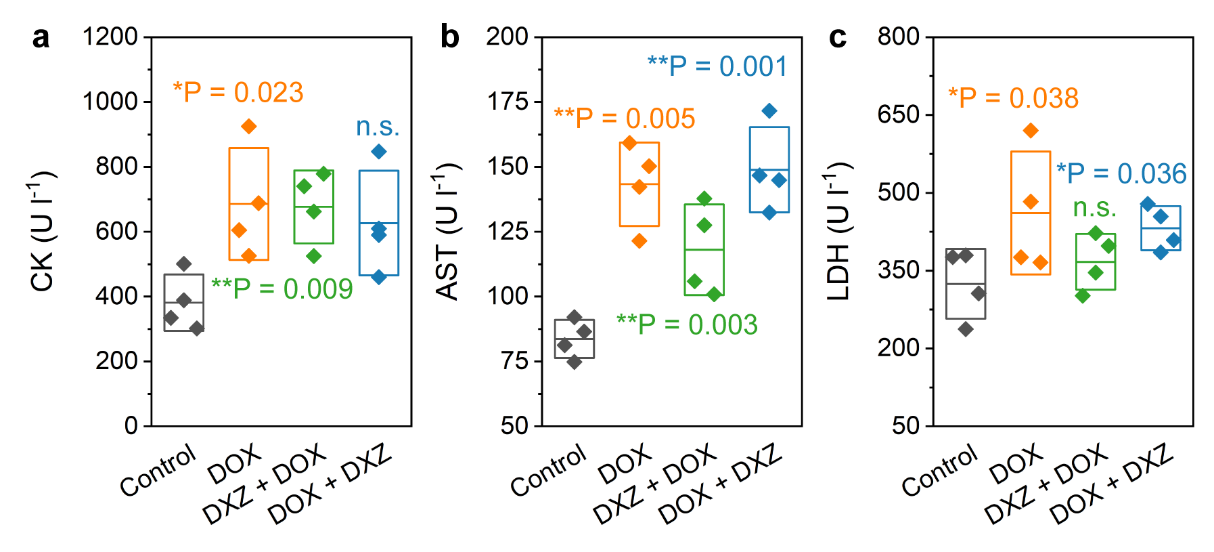


**Supplementary Figure 37.** Plasma CK (**a**), AST (**b**) and LDH (**c**) levels of each mouse in different groups at the end of the evaluation period. n = 4. Data are presented as mean ± s.d. Statistical significance (to the control group) is assessed by Student t’s two-tailed test. *P < 0.05, **P < 0.01 and n.s. for non-significant. Data are presented as they are and mean (line) ± s.d. (bounds of box).


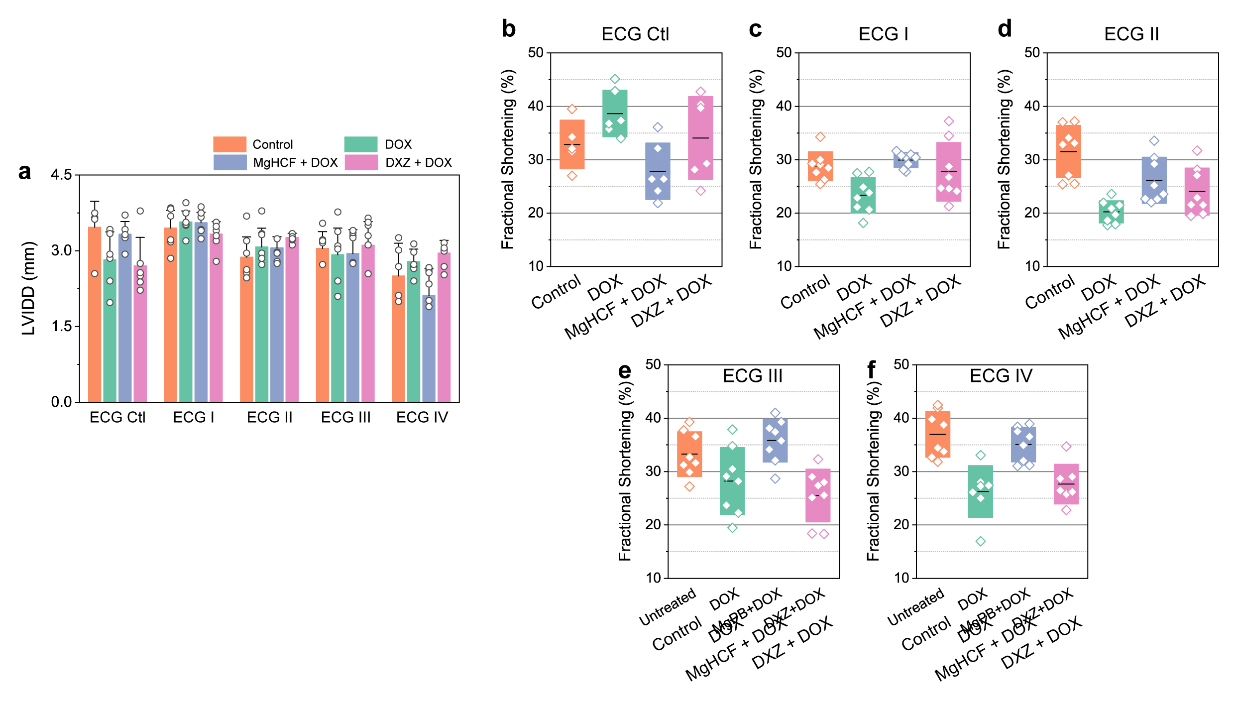


**Supplementary Figure 38.** **a-f**, Left ventricular internal diastolic diameter (LVIDD) profile (**a**) and LVFS profile (**b-f**) for mice treated with different groups at different ECG inspections. ECG Ctl (n = 6); ECG I (n = 8); ECG II (n = 8); ECG III (n = 8); ECG IV (n = 8). Data are presented as they are and mean (line) ± s.d. (bounds of box).


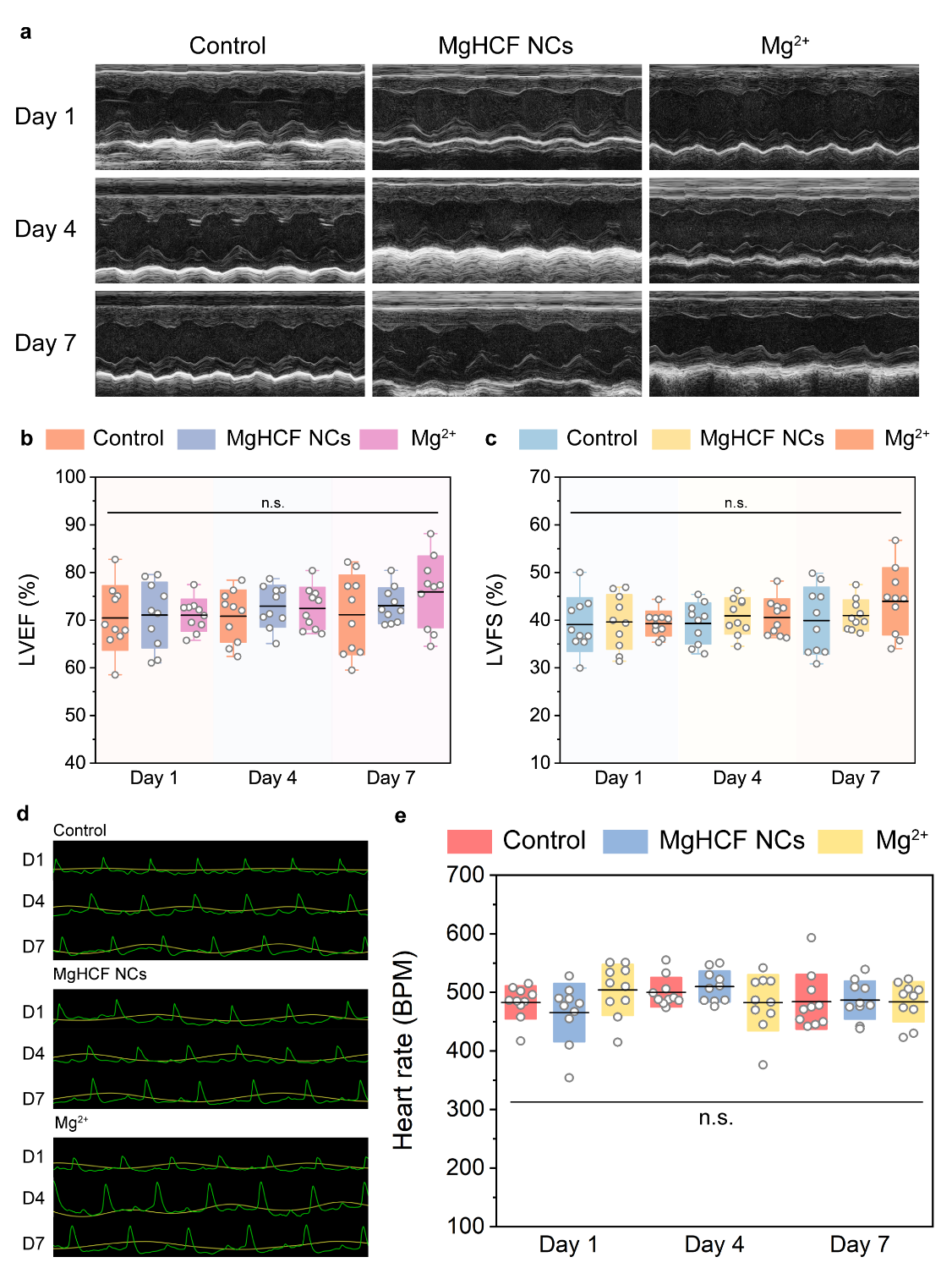


**Supplementary Figure 39.** **a**, Representative echocardiographic images of mice in different groups by echocardiography inspections. **b-c**, LVEF and LVFS data of mice in different groups by echocardiography inspections in Day 1, Day 4 and Day 7 of the biosafety evaluation. **d**, Representative Electrocardiogram images of mice in different groups by echocardiography inspections. **e**, Heart rate distributions of mice in different groups. n = 10. Data are presented as they are and mean ± s.d. Statistical significance is assessed by Student t’s two-tailed test. n.s. for non-significant.





**Supplementary Figure 40.** Survival experiment of MgHCF NCs administration intraperitoneally with 30 mg kg^-1^, 15 mg kg^-1^, 10 mg kg^-1^ and 7.5 mg kg^-1^, respectively (n = 10).


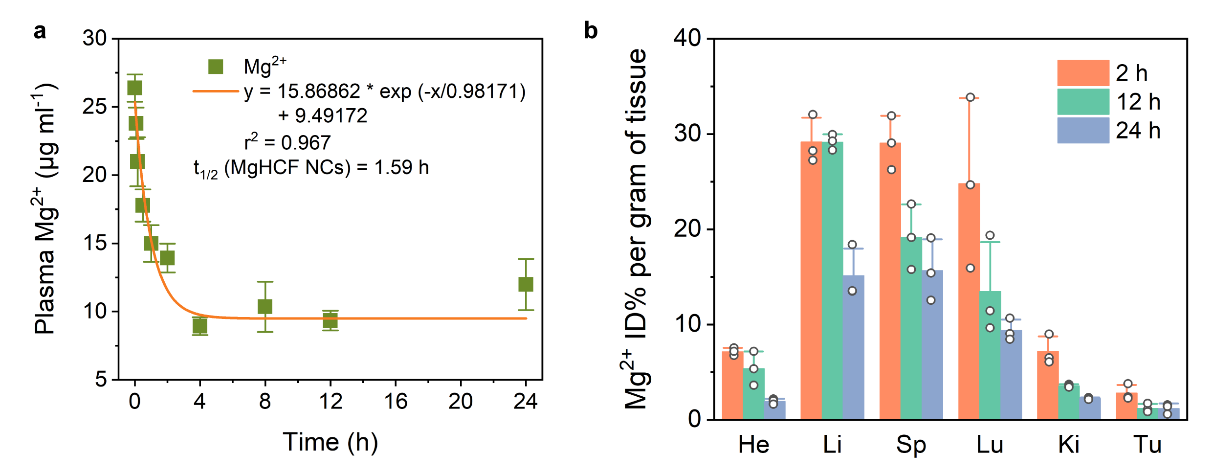


**Supplementary Figure 41.** **a**, In vivo plasma circulation of Mg^2+^ from MgHCF NCs upon administration in 24 h (n = 9 of biological replicates). **b**, In vivo biodistribution of MgHCF NCs to major organs upon administration in 2 h, 12 h and 24 h respectively (n = 3 of biological replicates). Data are presented as they are and mean ± s.d.





**Supplementary Figure 42.** Relative tumor inhibition rate profile for mice with different treatments during the therapeutic period.


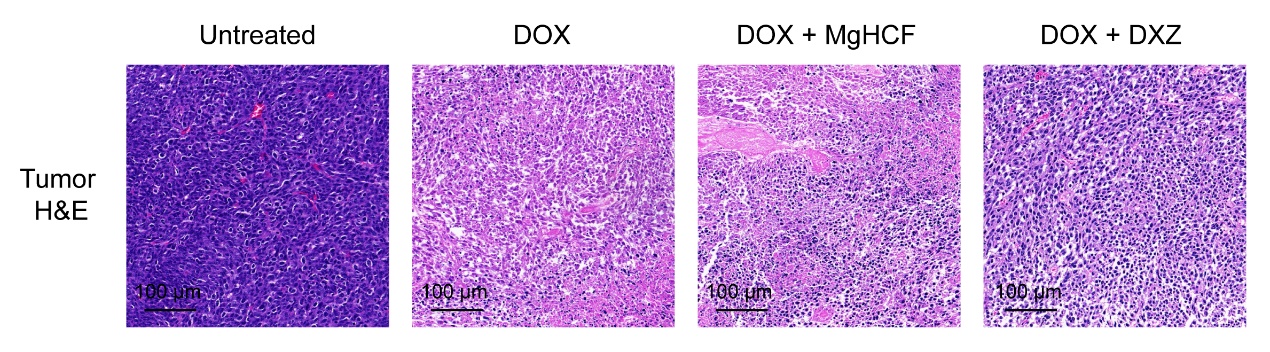


**Supplementary Figure 43.** H&E staining tumor sections of mice from different groups (Untreated, DOX, DOX + MgHCF, DOX + DXZ groups) after the in vivo tumor therapeutic period.
